# The effects of sediment properties on the aeolian abrasion and surface characteristics of microplastics

**DOI:** 10.1098/rsta.2024.0443

**Published:** 2025-10-23

**Authors:** Lucrecia Alvarez Barrantes, Joanna Bullard, Samuel Davis, Cheryl McKenna Neuman, Patrick O'Brien, Paul Roach, Zhaoxia Zhou

**Affiliations:** ^1^ Geography, Loughborough University Faculty of Social Sciences and Humanities, Loughborough, UK; ^2^ Geography and Environment, Loughborough University, Loughborough, UK; ^3^ Loughborough Materials Characterisation Centre, Loughborough University, Loughborough, Leicestershire, UK; ^4^ Trent Environmental Wind Tunnel, Trent University, Peterborough, Ontario, Canada; ^5^ Loughborough University School of Science, Loughborough, UK

**Keywords:** microplastic, aeolian abrasion, sediment shape, particle velocity, wind erosion

## Abstract

Microplastics (less than 5 mm diameter) are significant environmental contaminants whose small sizes and low densities facilitate transport by wind. Transport by wind erosion alongside soils or sediments results in mechanical abrasion of the plastic surfaces which can alter their physical and chemical properties. This paper uses laboratory simulations to determine the effects of up to 216 h of aeolian abrasion on polyethylene microplastics by angular, sub-rounded and rounded mineral sediments. During the abrasion process the mineral particles break down producing small fragments which adhere to the microplastic surfaces altering their surface roughness and chemistry. With increasing duration of abrasion the microplastic surface becomes coated with mineral fragments changing the dominant surface element from carbon to oxygen and silicon reflecting the composition of the erodents. The coating develops more rapidly when microplastics are abraded with angular sediments as these produce a lot of small fragments within the first 1–2 h. However, after more than 200 h of abrasion all the erodents had similar effects. A conceptual model of microplastic surface change is presented in which the plastic cracks and fractures, then flattens along with increasing density of sediment fragment cover. Surface changes may affect the ability of the plastics to transport airborne contaminants.

This article is part of the Theo Murphy meeting issue ‘Sedimentology of plastics: state of the art and future directions’.

## 1. Introduction

Microplastics are synthetic polymer-containing particles that can be directly produced by the plastics industry (primary microplastics) or formed through fragmentation of larger plastics (secondary microplastics) [1]. While numerous classifications of plastic size have been proposed, microplastics are typically categorized to be in the range 1 µm to 5 mm, while nanoplastics are less than 1 µm [2]. Microplastics are present as contaminants in the environment and can affect the functioning of abiotic and biotic systems by changing their physical properties as well as acting as a pollutant [3].

The small size and low density of many microplastics means they are typically present on the surface of soils and sediments due to their enhanced susceptibility to redistribution alongside mineral particles by fluvial, aeolian and marine processes [4–7]. These processes can also change the physical and chemical properties of microplastics through interaction with other particles or surfaces, and by exposure to ultraviolet light from solar radiation, which results in plastic weathering (ageing) [8–12]. The further environmental cycling of microplastics through repeated entrainment, transport and deposition identifies them as a potentially important component of Earth’s global carbon cycle [13,14].

One of the least well studied components of plastic cycling is its entrainment into the atmosphere. Unmanaged macroplastics (e.g. plastic bags) can be entrained by the wind and transported long distances from source resulting in litter pollution and potential harm to fauna [15–17]. Near-surface aeolian transport can cause macroplastics to wear through mechanical abrasion as they collide with, or are dragged by the wind across both natural and anthropogenic surfaces including those that are consolidated (e.g. bedrock, concrete, tarmac) and unconsolidated (e.g. sandy beaches or deserts, agricultural soils) [18]. Microplastic particles within sediments, for example those associated with the surface application of contaminated sewage [7,19], the degradation of agricultural plastic mulch [20], or those resulting from long-distance transport and deposition [21], can be entrained and transported alongside mineral particles by wind erosion [22].

Physical and chemical changes to plastics can be caused by photodegradation, but are also expected to occur due to mechanical wear particularly as, in many cases, the material from which the microplastic is composed will be softer, less dense and more susceptible to alteration than mineral particles [20,23]. It is well known that collision and thus abrasion during the transport of mineral particles by wind can alter their shape, size and surface chemistry (e.g. [24–27]). The coincident transport of airborne mineral and microplastic has further been observed to change the properties of plastic particles [12]. These changes may affect plastic interaction with its surrounding environment, leading to important differences in transport and accumulation.

Understanding the effects of aeolian transport and associated abrasion on microplastic properties is important if the latter are to be effectively and accurately incorporated into atmospheric models to identify possible sources of microplastic pollution and predict atmospheric transport pathways [28,29]. Research into the aeolian abrasion of mineral particles has determined that properties such as particle size, sorting, shape, lithology and roundness affect the rate of mechanical breakdown and resulting characteristics of the initial (parent) particles as well as the quantity and size of the products of abrasion [24,30,31]. These properties affect variables such as kinetic energy (KE) transfer between individual particles. It is expected that the aeolian abrasion of microplastics during wind erosion would be affected by the properties of not only the plastic, but also the mineral particles (erodents) alongside which they are entrained.

The aim of this paper is to determine the effects of erodent properties on the aeolian abrasion of microplastics. This is achieved through laboratory experiments using combinations of three different mineral sediments (erodents) and polyethylene spheres (microplastics). The erodents differ in both size and shape, which in turn are expected to affect particle velocity and consequently the energy with which they interact with microplastics and potentially change microplastic size and properties. The relative size of mineral particles and microplastics is also expected to affect their interaction, and to examine this, three different sizes of microplastic spheres were tested with each erodent. Abrasion of the microplastics is quantified by examining changes to the microplastic size, surface chemistry and surface texture.

## 2. Methods

### (a) Erodents and microplastics

A series of experiments was conducted using three types of mineral particles (sediment) and three sizes of microplastic (table 1), resulting in nine combinations of erodent and microplastic. The microplastics (Mp) were pristine, fluorescent, proprietary polymer (Cospheric, California, USA) microbeads comprising ≥ 70% polyethylene by weight. Polyethylene was used because this is one of the most common polymer types found in surface soils [33,34] and, although there are ongoing debates around the relevance of microplastic experiments using pristine microplastics [35,36], the advantage of using these is confidence that experiments and replicates were comparable in terms of the initial material and shape of the microbeads [37]. In the natural environment microplastic particle counts are highly variable with a recent review of microplastic in soils presenting counts from 0 particles kg^−1^ to over 3 000 000 particles kg^−1^ [38]. The majority of studies report counts of < 10 000 particles kg^−1^ but many of these exclude particles less than 0.5 mm. In this study, for all experiments, 10 g of erodent and 0.01 g of Mp were added to an abrasion chamber. Using these relative proportions, the equivalent particle count per kg is 4025 for the largest microplastics used (Mp_710_), 32 196 for Mp_355_ and 1 29 118 for Mp_212_. The lower counts kg^−1^ of microbeads used in this study are comparable with counts obtained in many agricultural settings and the higher counts (greater than 100 000 particles kg^−1^) are more representative of urban or suburban soils [39,40] or soils associated with landfill,or where biosolids containing microplastics have been applied [41]. Prior to use, the sediment was dry-sieved to remove particles less than 250 μm and greater than 500 μm and washed to remove any adhering fine particles. The detailed particle size characteristics listed in table 1 were determined using a Malvern Mastersizer 3000 which uses a volume-based calculation (assuming particles are spherical). As a consequence, there is an expected discrepancy between the sieved sizes (250–500 μm) and the reported D_10_, D_50_ and D_90_ values (which can exceed 500 μm) due to differences in the measurement methods (e.g. [42]; [43]). For each combination of sediment-microplastic, a separate experiment was run for six different abrasion times: 1, 2, 24, 72, 144 and 216 h. Abrasion times were chosen based on results from previous studies where most particle changes, including to surface chemistry, occur within the first 72 h [12,31,44], but recognizing that longer periods are more representative of residence times for particles in the natural environment [25,26].

**Table 1.** Sediment and microplastic properties used for the abrasion experiments.

name	description	size (μm)			circularity	ratio (D_min_/D_Max_)	density (kg m^−3^)	roundness [32]
		D_10_	D_50_	D_90_				
ROU	rounded commercial sand	266	345	448	0.734	0.774	2650	0.70 ± 0.07
SUB	subangular sand	242	319	422	0.738	0.752	2650	0.53 ± 0.14
ANG	angular sand	291	414	528	0.610	0.712	2500	0.07 ± 0.03
Mp_212_	microplastic polyethylene sphere	212−250			0.920	0.956	1200	—
Mp_355_	microplastic polyethylene spheres	355−425			0.923	0.961	1000	—
Mp_710_	microplastic polyethylene spheres	710−850			0.900	0.960	1000	—

### (b) Abrasion chamber

**Figure 1.**
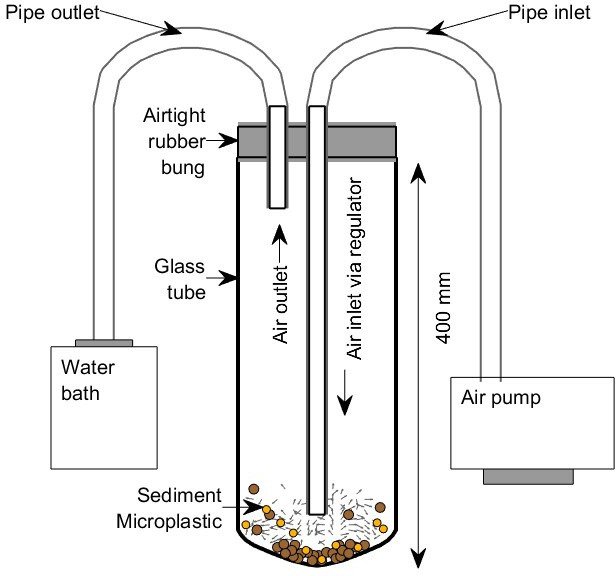
Test-tube chamber used to run the abrasion experiments for the microplastic and sediment combinations.

The aeolian abrasion process was simulated using a widely used test-tube chamber design [12,31,44,45] shown in figure 1. The main structure consists of a glass tube 100 mm in diameter and 400 mm high, with a conical base to promote particle circulation. A jet of air with constant flow velocity (14 m s^−1^) is fed to the base of the chamber using a pump. This inlet flow velocity cannot directly be equated to wind speeds in the natural environment due to being confined within the glass chamber, but particle velocities were measured, as detailed below, to ensure the impact velocities and KE were comparable with those achieved in natural saltation clouds. Deflection of the airflow off the glass wall produces a recirculation cell with weak vortical motion that agitates the particles inside the chamber. Particle movement and associated particle–particle collisions simulate processes of saltation, creep and resulting abrasion during wind erosion [31]. The particles inside the chamber are abraded for defined periods of time following which the source materials and products of abrasion are collected for analysis. To minimize electrostatic charging of the microplastics, unlike in some other studies, an electrostatic precipitator is not used with the chamber and instead the outlet tube is connected directly to a water bath containing deionized water which collects any suspended particles created and/or released during the abrasion process.

Alongside the particle characteristics, two key factors determining the rate of abrasion are the particle velocity and mechanical energy available for abrasion within the chamber. For this study, particle velocity within the chamber was measured and then used to estimate energy. Particle velocities were measured using a Dantec™ 2D laser doppler Anemometer (LDA)—the FlowExplorer—mounted on a two-dimensional (2D) programmable traverse. The LDA was configured to measure the velocity of particles using a 2D grid bisecting the centre of the chamber. The node resolution within the grid is 5 mm and extends from −50 to +50 mm in the *x*-axis and from 0 mm (lowest point of the chamber) to 80 mm (top of the particle cloud) in the *y*-axis (electronic supplementary material, fig. S1). At each *x*_*i*_–*y*_*i*_ node within the grid, the laser was configured to detect all particles that passed through the LDA sample volume for a period of 5 s or until 1500 particle counts had been obtained. The resulting dataset provides information about the particle horizontal velocity (Uv m s^−1^), vertical velocity (Vv, m s^−1^) and intensity of the laser signal (mV). Importantly, the LDA is unable to differentiate between particle types within a mixed population, therefore the particle velocity values obtained in this study are not binned by diameter and material type.

The dataset was filtered to remove anomalous values in three steps. First, any readings where the intensity return was more than ± 2 standard deviation (*σ*) from the median intensity were discarded. The intensity parameter is correlated with particle diameter and extreme values can be associated with unrealistic measurements [46]. Second, measurements were also removed if the velocity values fell more than ± 2 *σ* from the median velocity ($U=\sqrt{{Uv}^{2}+{Vv}^{2}}$). Finally, any grid nodes with fewer than three particle velocity measurements remaining were removed as this was considered an insufficient sample size for robust analysis. The quantity of particle velocity measurements removed from the analysis by this filtering process ranged from 6.7% to 10.2% for a run with the lowest remaining number of measurements being 29 688 (for ANG-Mp_212_).

The mechanical energy (*E*, J Kg^−1^) per unit mass of particles circulating within the chamber, and hence the potential for particle abrasion, can be approximated using the fundamental continuity relation,


(2.1) $$E=\frac{1}{2}{\bar{U}}^{2}+gH.$$

This summation of the kinetic (KE) and potential energy (PE) for the cloud of suspended particles is simplified to consider only the mean particle velocity (*Ū*, m s^−1^) within the chamber and the cloud height (*H*), which is a common metric reported in aeolian transport studies. Herein, *H* was estimated as the median *y*-value of the 90^th^ percentile of the particle count-rate (number of particles detected in a defined period of time) at each *x*-value. Gravitational acceleration is given as *g* (m s^−2^) in equation (2.1). A summary of the particle velocity, saltation cloud and energy values achieved for most combinations of sediment and microplastic tested is listed in electronic supplementary material, table S1. There was insufficient mass of the ANG sediment sample remaining to conduct velocity tests for all microplastic sizes, so no particle velocity or energy data are available for ANG-Mp_355_.

### (c) Characterization of abraded samples

Following each experiment, the sediment-microplastic mixture remaining in the chamber was examined using a field emission scanning electron microscope (SEM)—(JSM-7800F, JEOL Limited, Tokyo, Japan)—operating at a 5 kV electron accelerating voltage. All samples were sputter-coated (Quorum Q150T, Quorum Technologies, UK) with an approximately 10 nm thick gold/palladium layer prior to analysis to limit surface charging. Secondary electron images were recorded using the SEM to measure the change of the microplastic particle dimensions and their surface texture/microstructure as a function of abrasion time. The SEM was also equipped with energy-dispersive spectroscopy, which was used to analyse the chemical composition and map the elemental distribution at the surface of the microplastics after each abrasion experiment.

The contents of the water bath were filtered through a 0.45 µm cellulose nitrate membrane filter. Drying and weighing the filter papers enabled quantification of the mass of fine particles created during the abrasion period. The mass of the fragments includes both mineral particles and microplastic or nanoplastic particles created by abrasion but due to the very small total quantities produced (less than 0.01 g for most experiments) it was not possible to accurately separate the two material types by weight. In line with other studies, the mass of fine particles (sediment + plastic) produced is expressed as a % of the initial sample weight (%ISW).

## 3. Results and discussion

### (a) Erodent characteristics and fragment production

Fine particles were produced by abrasion for all experiments and these are expected to comprise a mix of mineral and plastic fragments. For this study, the rate of mineral particle abrasion, and production of fine mineral particles, is pertinent because this process affects both the shape of the parent particles (increased abrasion causes rounding of mineral particles) and the material in the chamber that is available to interact with the microplastics.

**Figure 2.**
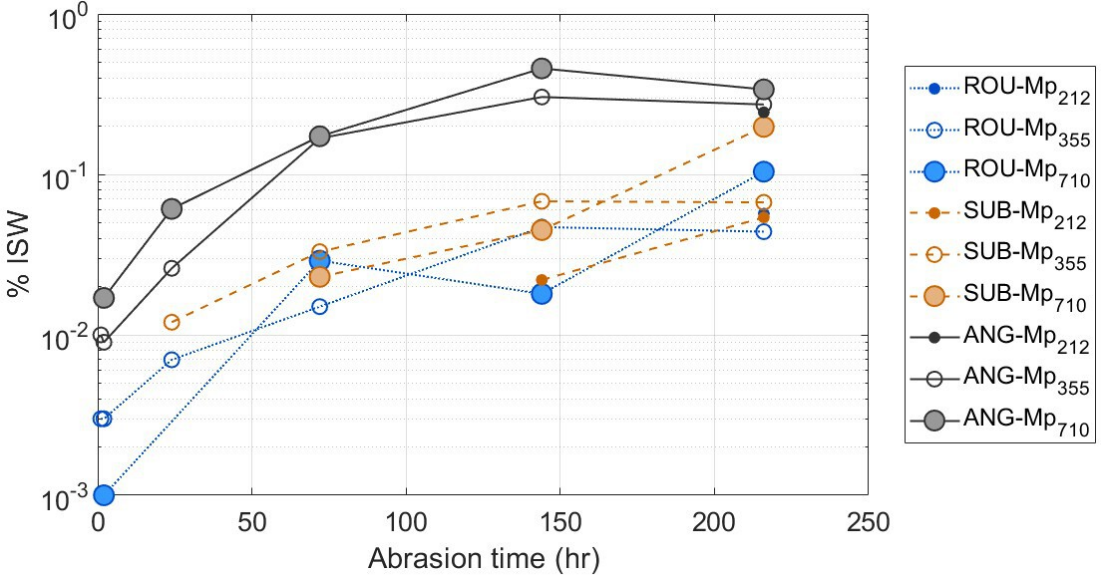
Total sediment and microplastic fragments produced from each sediment-plastic mix expressed as a percentage of the initial sample weight (%ISW).

Over the maximum 216 h abrasion time, the total fragment mass produced ranged from 0.05 to 0.34% ISW (figure 2). There are few other studies that have reported values for greater than 200 h abrasion, but after 72 h the material abraded here had produced fragments weighing 0.02–0.17% ISW which is within the range of values for most other 72 h experiments (0.005 to 2.3 %ISW [30,31]), for sub-angular to sub-rounded sands. The values here may be slightly lower because, without the electrostatic precipitator, collection efficiency is likely to be reduced [31]. The microplastics only contribute less than 0.1% by weight to the mass of material in the chamber and are not expected significantly to affect the mass of fragments on the filter papers. However, in all cases abrasion of the sediment with larger microplastics (Mp_710_) results in a greater quantity of total fragments by weight than when smaller microplastics are used.

**Figure 3.**
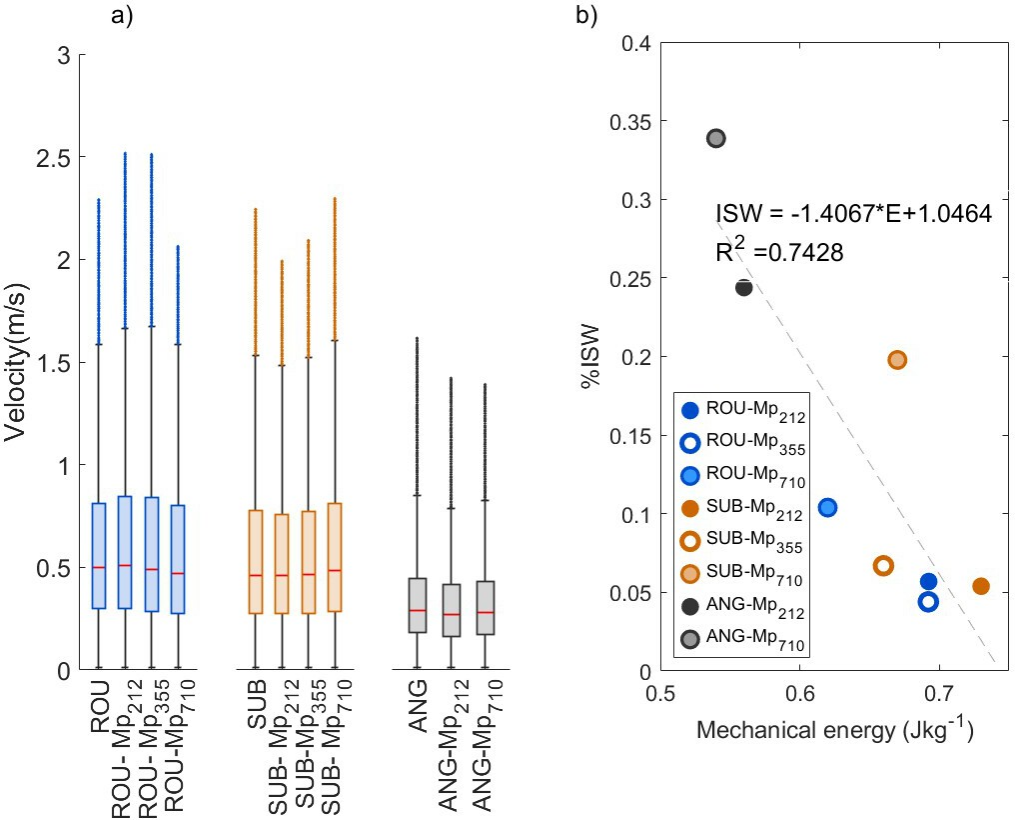
(a) Distribution of particle velocities for different sediment-microplastic combinations. Boxplot shows median, 25^th^ and 75^th^ percentiles of velocity data. Values greater than 1.5 times the interquartile range are shown as dots (typically velocities greater than 1.5 m s^−1^ for ROU and SUB, and greater than 0.8 m s^−1^ for ANG) (b) Relationship between mechanical energy within the abrasion chamber and the mass of fragments produced as %ISW after 216 h abrasion.

Within the abrasion chamber, particles with different sizes and shapes are expected to achieve different particle velocities for the same airflow input [31]. This in turn will generate different amounts of mechanical energy and hence abrasion potential [47]. As the concentration of microplastics is very low in the chamber, it is assumed that the particle velocity distribution is dominated by velocities achieved by sediment particles (rather than microplastics). For the three experimental groups presented here, sediment-microplastic combinations containing ROU and SUB have very similar positively skewed velocity distributions with modal particle velocities at 0.325 m s^−1^ and 0.3 m s^−1^ respectively, and mean particle velocities of 0.47−0.5 (ROU) and 0.46−0.49 m s^−1^ (SUB) (figure 3*a*). By contrast, the modal particle velocity for ANG is 0.175 m s^−1^ and the mean particle velocity 0.28−0.3 m s^−1^ (electronic supplementary material, table S1). These velocity distributions and modal values are very similar to those measured in natural saltation clouds which typically are less than 1 m s^−1^ close to the bed and in the range 2−4 m s^−1^ at 20−40 mm above the bed [48–50]. For the sediment-microplastic combinations ROU-Mp_212_ and ROU-Mp_355_, there is a substantial increase in the number of particles (> 1500) with measured particle velocities greater than 2 m s^−1^ in the samples containing microplastics compared to those without, which may be attributable to the presence of the microplastic particles.

The standardized pressure energy provided to the closed system enabled particle uplift via the deflected air jet. The saltation cloud heights (approximated from the vertical displacement of 90% of particles sampled) are very similar between experiments, ranging from 49−54 mm for sediment-microplastic combinations with ANG, to 50−56 mm for those with SUB and 46−50 mm for those with ROU. Variation in E (equation (2.1)) would appear therefore to be predominantly driven by that associated with KE, and specifically, $\bar{U}$. Partitioning of this energy occurred during collision with the glass walls and neighbouring particles, so that particle surface deformation, fracturing and chipping (spalling) all involved varied amounts of mechanical energy (*KE*) loss within the chamber. As expected, figure 3*b* reveals an inverse relationship between the mass of fragments produced and E for each sediment-microplastic combination. This general trend is modified, however, by particle size and shape. For similarly shaped particles, for example, particle velocity might be expected to decrease with decreasing particle size. For a standardized quantity (by mass) of particles inserted in the chamber, the probability of particle collision, material fatigue and energy loss is expected to be higher for particles which are smaller in diameter and thus more numerous.

An exception to this rule concerns the erodent ANG, which has a high rate of fragment production even though the sample contains particles that are relatively coarse. The observed effect of shape on the overall mass of fragments produced through time (figure 2) accords with previous reports that abrasion rates are higher for angular particles than rounded particles [24,25,51,52]. Angular particles are more susceptible to chipping and spalling because the asperities are subjected to a disproportionately large concentration of the impact energy [53]. When angular particles are abraded, the rate of fine particle (fragment) production typically is rapid in the first few hours of abrasion and then slows down with abrasion and smoothing of the particle surface [44,54], which was similarly observed for ANG. By contrast, the sub-angular and rounded particles generate fine particles relatively slowly during the first 100 h but fragment production increases towards the end of the experiment (150+ h). Smith *et al.* [53] attribute this temporal trend to fatigue; that is, fracture arising from the cumulative effects of stresses imparted by multiple collisions as opposed to rare but highly energetic collisions (e.g. involving only a few particles with large mass). An additional contributing factor is the relative hardness of the ANG sediments, which is lower (900 HV) than for quartz (1200 HV) and may have contributed to greater amounts of fragment production.

### (b) Effect of abrasion on microplastic size

**Figure 4.**
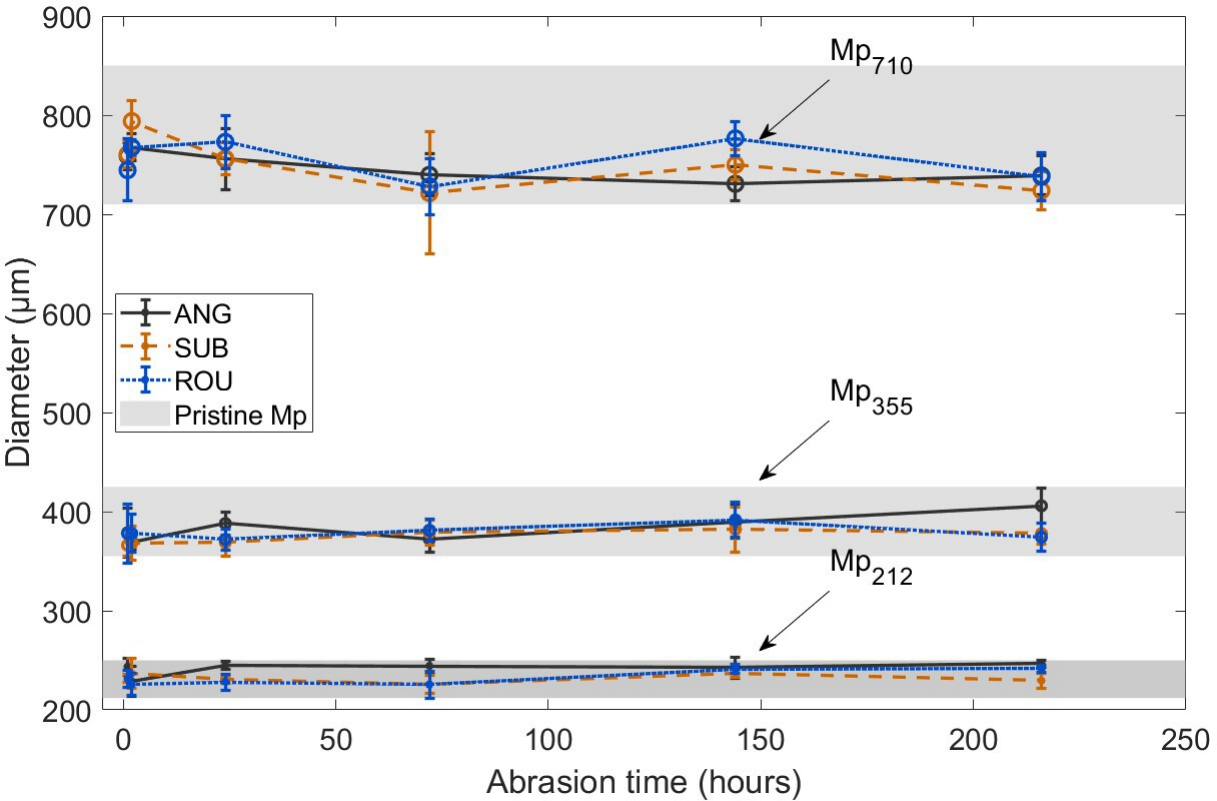
Change in microplastic diameter after different durations of abrasion. Values are mean diameters (µm ± 1 *σ*). The grey shading indicates the size range of unabraded microplastics from table 1.

The measured sizes of the microplastics after abrasion with different sediments are presented in figure 4. It is not possible to track individual microplastic spheres to examine the same particle before and after abrasion, consequently particles are sampled and the mean diameter ± 1 *σ* shown. Even after 216 h of abrasion, in all cases the sampled microplastic spheres are within the range of sizes expected without abrasion, as shown in figure 4 (table 1). This suggests that no systematic change in Mp size has taken place during the abrasion process. Some previous studies have documented a reduction in microplastic size with mechanical abrasion [12] but this is not universal, for example, Song *et al*. [8] abraded low density polyethylene pellets in a tumbler for two months with sand and found that, in the absence of photodegradation, there was no discernible change to the diameter of the plastic particles. Laboratory engineering research has identified that in the initial stages of polymer abrasion, an incubation period may occur in which little or no mass is lost, as impact energy is dissipated in reworking the target surface [55]. This can be associated with embedding of fragmented mineral particles into the plastic surface which then shields the surface from subsequent impacts reducing abrasion rate [56,57]. This type of change cannot be discerned from particle size measurements alone and requires detailed examination of the microplastic surfaces.

### (c) Microplastic surface characteristics

#### (i) Surface texture

**Figure 5.**
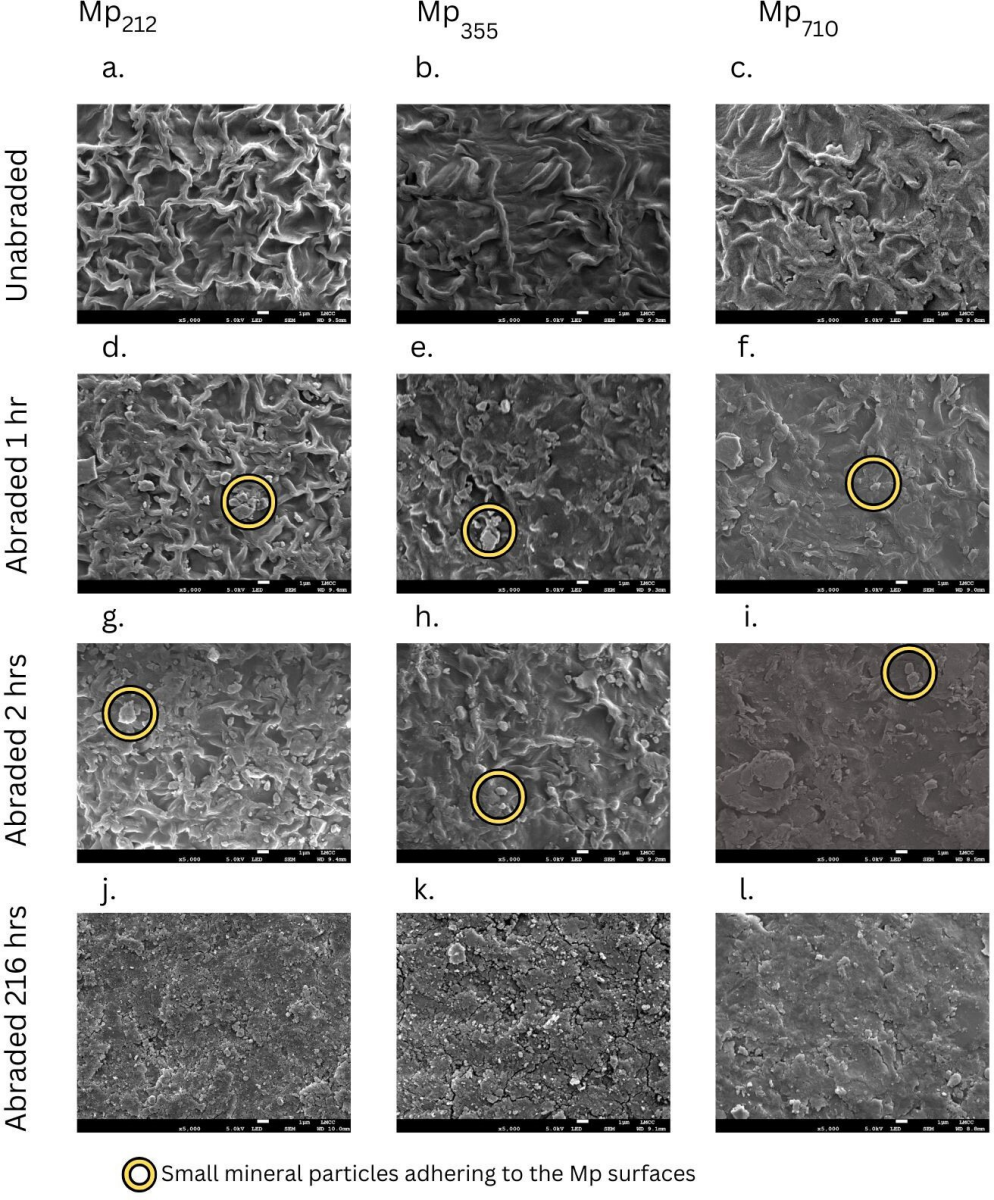
Representative SEM images of microplastic surfaces with a magnification of 5000x. (a–c) Unabraded plastics. Subsequent panels show microplastic surfaces after abrasion with SUB for (d–f) 1 h, (g–i) 2 h and (j–l) 216 h. Scale bars indicate 1 µm. SEM images for microplastic abrasion with SUB for periods of 24 h, 72 h and 144 h are given in electronic supplementary material, fig. S4.

The microplastic surfaces underwent considerable visible physical changes following even short periods of abrasion (e.g. 1 h). This is illustrated in figure 5 using SEM micrographs for plastics abraded with SUB and the changes were similar for abrasion with ANG and ROU (electronic supplementary material, figs. S2, S3). The pristine microplastic surface morphology comprises an evenly distributed labyrinthine, rugose surface formed by rounded, intersecting ridges approximately 1 µm in width (figure 5*a*–*c*). After 1 h of abrasion the ridges are less well-defined and small particles adhering to the Mp surface are visible (figure 5*d*–*f*). The smallest fragments are less than 2 μm diameter. With increasing duration of abrasion, the polymer ridges become progressively less distinct, and the number of adhering particles increases until after 216 h abrasion the ridges are no longer discernible and the adhering fragments cover most of the microplastic surface (figure 5*j*–*l*). Cracks also become visible on the particle surfaces. The flattening of the microplastic ridges appears to occur more rapidly on the larger Mps compared with the smaller ones but the surfaces of all the microplastics appear very similar after the maximum period of abrasion (figure 5).

The formation of a physical coating of mineral fragments on microsphere surfaces has been described by Bullard *et al.* [12], who suggested these are formed by the adhesion of small mineral particles created during the abrasion process to the plastic. The longer the period of abrasion, the thicker the coating [12]. The same phenomenon has been observed in sediment particles where nano-scale mineral fragments adhere to the surfaces [58].

#### (ii) Microplastic surface composition

**Figure 6.**
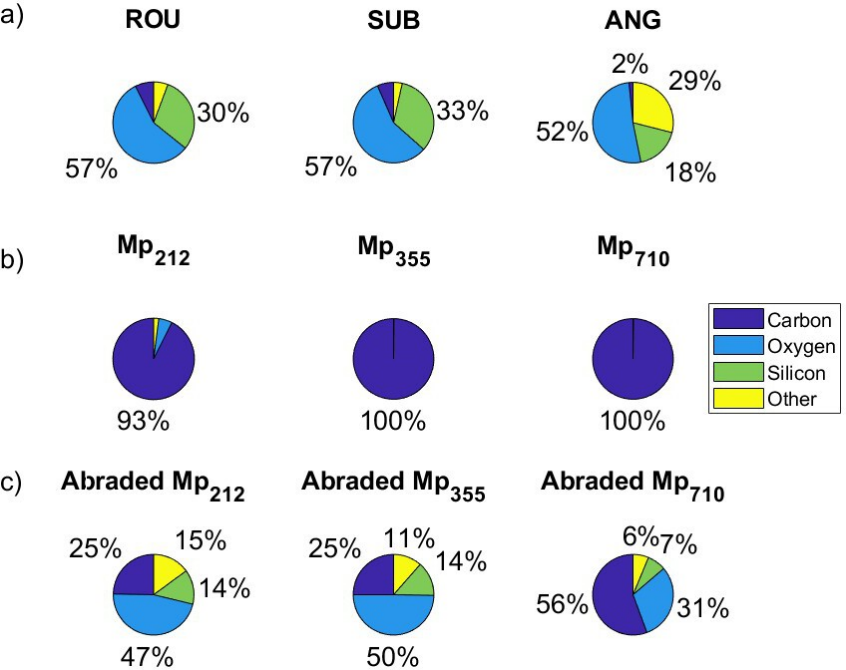
Summary of the sediment and microplastic surface chemistry composition: (a) unabraded sediment; (b) unabraded microplastic, (c) microplastic after 216 h of abrasion.

Analysis of the surface atomic composition of the microplastics can determine whether the small particles adhering to the microplastic surface comprise fragments of plastic or fine mineral particles. The surface atomic composition of the erodents (detailed in electronic supplementary material, table S2) is summarized in figure 6*a* and demonstrates the dominant elements are carbon (C, 6–7%), oxygen (O, 57%) and silicon (Si, 30–33%) for ROU and SUB while ANG also includes notable quantities of iron (11%), aluminium (6%) and calcium (5%). The microplastic surfaces are dominated by carbon (92.6–99.9%). Following 216 h of abrasion, the surface composition of the microplastics was more varied. For Mp_212_ and Mp_355_, surface carbon composition decreased from more than 93% to 25% and oxygen became the dominant element (47–50%) along with a substantial amount of silicon (14%). Even after abrasion, the surface of the large Mp_710_ remained dominated by carbon but the percentage coverage decreased from 100% to 56% and the carbon was replaced by oxygen (31%) and silicon (7%). Given the composition of the erodents, the most likely cause of this change to the microplastic surface composition is that fine particles broken from the erodents during abrasion have adhered to the plastic, altering their surface chemistry.

**Figure 7.**
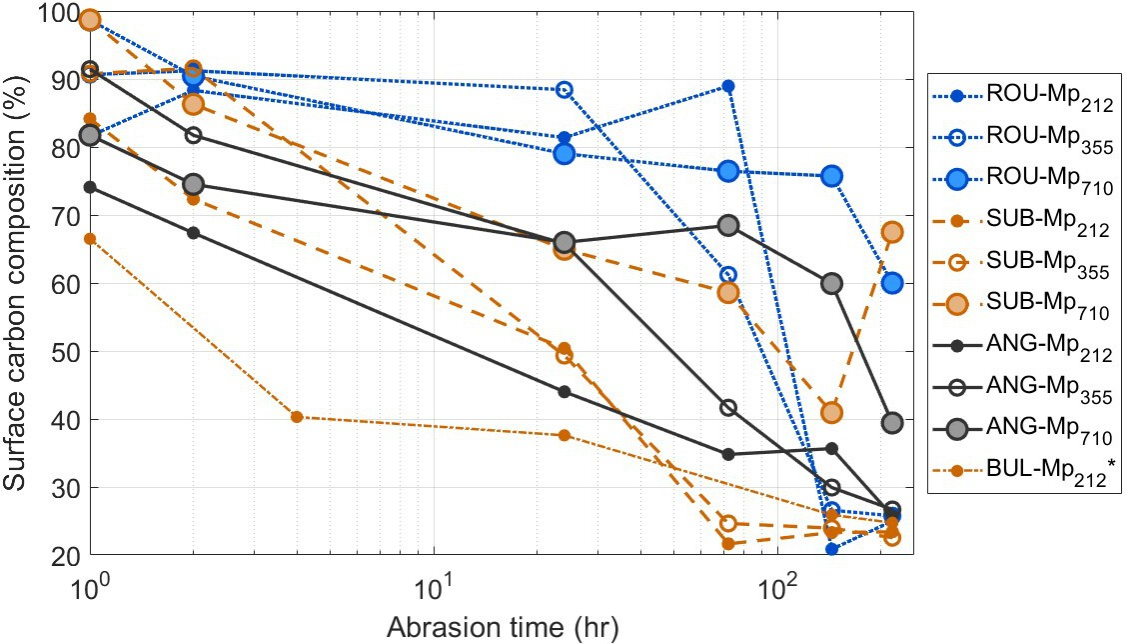
Change to the carbon content of the microplastic surfaces with increasing abrasion time. Data for BUL-Mp_212_ are taken from [12].

The rate at which the coating of fine mineral particles on the microplastic surface develops varies with abrasion time, the nature of the erodent, and the size of the microplastic (electronic supplementary material, table S2). For example surface chemistry changes most rapidly in the first 72 h when small and medium microplastics are abraded with angular particles, but it is in the period from 144 h to 216 h when the large microbeads are abraded. Surface chemistry changes are most rapid in the first 72 h for abrasion with subangular particles, regardless of microplastic size. By contrast, for abrasion using rounded particles the most rapid period of microplastic surface chemistry change is between 72 h and 144 h for small and medium microbeads, but within the first 24 h for large microbeads. This is illustrated in figure 7 by focusing on the change to the percentage of carbon at the microplastic surface. The microplastics abraded with ANG registered a rapid reduction in surface carbon (from 92.6–99.9% to 76.86%) after only 1−2 h of abrasion following which the decrease in carbon is slower but consistent. By contrast, the surface carbon of microplastics abraded alongside ROU remains greater than 75% for the first 24 h and then for Mp_212_ and Mp_355_ drops rapidly from more than 60% at 72 h to less than 30% at 144 h. For the small and medium microplastics, with all erodents, the proportion of carbon remaining detectable on the bead surface is less than 30% after 216 h of abrasion. For the Mp_710_ the reduction in surface carbon takes longer suggesting the mineral coating takes longer to develop and cover the whole bead. At the end of the abrasion period the large microplastics abraded with sub-angular and rounded erodents still have more than 60% carbon at the surface. The results of one of the samples used by Bullard *et al.* [12] is included in figure 7 (BUL-Mp_212_), showing a similar trend to that observed here for sub-angular particles, i.e. a rapid reduction in surface carbon within the first few hours of abrasion followed by a gradual decrease throughout the remainder of the abrasion experiment.

## 4. Combined effects of abrasion on microplastic surface texture

All of the erodents affect the surface properties of the microplastics. The more angular erodent (ANG) caused the most rapid change to the microplastic surface texture; however, for this type of plastic after more than 200 h of abrasion there was no discernible difference in microplastic texture regardless of the erodent used. The progressive alteration of microplastic surfaces by mechanical impact has also been documented by Ren & Ni [20] who simulated wind erosion alongside agricultural soils and reported increased degradation of plastic film with longer duration of aeolian abrasion, and with higher wind speeds. While scanning electron microscopy is ideal for visualizing microplastic surface properties, it is limited in terms of quantitative analysis of surface roughness changes [59,60]. Ideally, a three-dimensional (3D) topographic survey of the plastic surface before and after abrasion using an approach such as atomic force microscopy would have been carried out [60,61] but the size and curvature of the microplastic beads tested here would present challenges for the maximum displacement of the measurement tip [62] and so it was not possible. Instead we use the SEM 2D images to propose a conceptual 3D model of changes to the microplastic surface.

**Figure 8.**
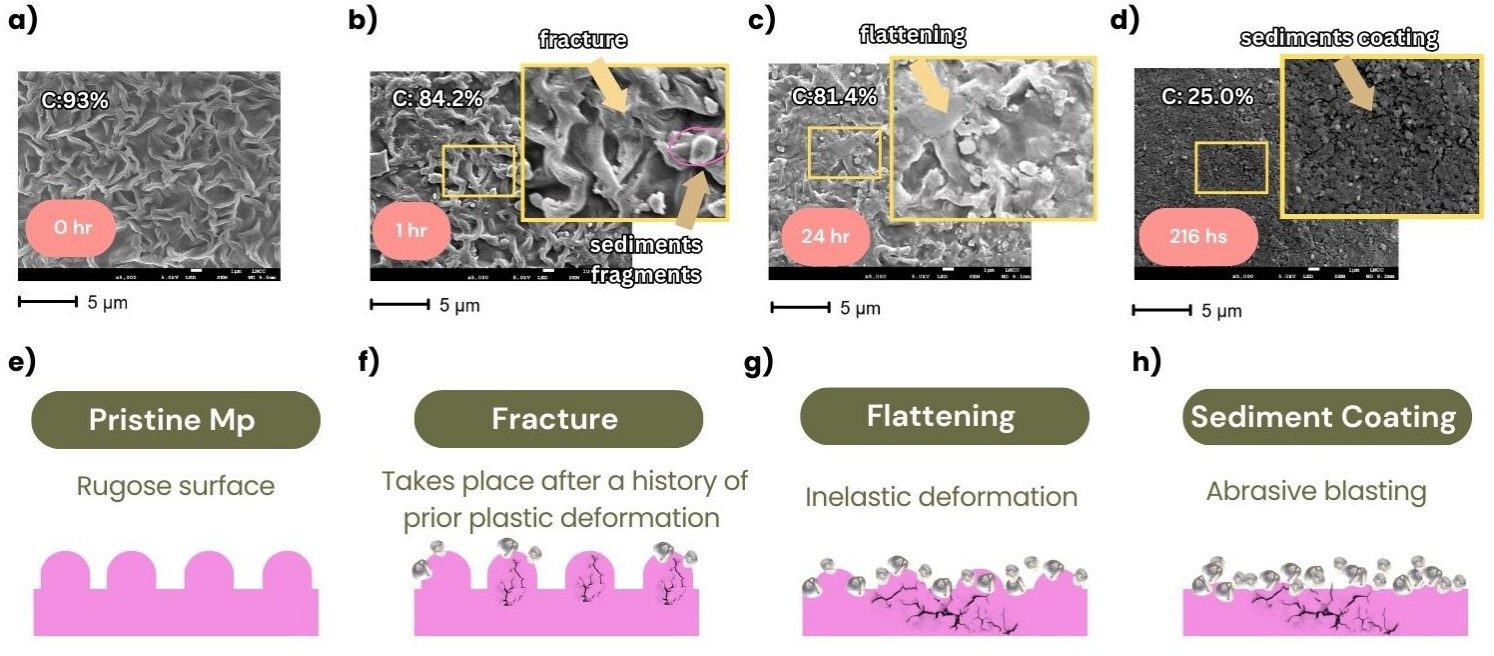
Conceptual model of changes to microplastic surfaces during the abrasion process, highlighting the mechanical adhesion of sediment fragments to the microplastic surface, change in surface carbon (C) and plastic surface failure, where fractures, flattening, and fragmentation of material occur. The SEM images are of microplastic surfaces from experiments SUB-Mp_212_ after (a) 0, (b) 2, (c) 24 and (d) 216 h. Diagrams (e–h) illustrate the process of sediment coating and plastic failure.

Progressive physical changes to the microplastic surface textures observed here are conceptualized in figure 8. Two main processes take place: mechanical adhesion of sediment fragments to the microplastic surface and failure of the plastic surface evidenced by fracturing, cracking and flattening. The mechanical adhesion of fragments, generated by breakdown of the parent mineral sediments, to the microplastic surfaces can be described as a microscale mechanical interlocking-anchoring of two surfaces [63,64]. This adhesion builds a sediment coating comprising small micro and nano particles (less than 1 µm) around the microplastic which is clearly visible using the SEM. Failure of the plastic surface starts to occur within the first few hours of abrasion, with visible cracks and fractures appearing at the surface. The process of flattening the ridges on the microplastic surface is an inelastic deformation which has been observed in other simulations of wind abrasion of mineral sediments [65] (cited by [66]) and microplastics [67]. Fracturing results from successive impacts between the microplastic particle and mineral particles, which cause surface fatigue leading to surface and subsurface cracking [68]. Other simulations of mechanical abrasion of microplastics with soil particles have identified multiple erosion signatures including fracturing, micro-ploughing and micro-cutting, which can also cause fragments of microplastic to become detached [69].

The balance between the effects of mechanical adhesion and plastic failure vary according to microplastic size. Mechanical adhesion is expected to increase or maintain the diameter of the microplastic particles whereas flattening and removal of plastic fragments following cracking, ploughing or cutting is expected to decrease their size. Bullard *et al*. [12] suggested that the sediment coating developed rapidly (during the first 24 h of abrasion) and was then maintained at a constant thickness equivalent to one fine sediment particle (1−1.5 µm) by repeated embedding and dislodgment of the sediments during which microplastic fragments could be removed, reducing the microplastic particle diameter. There is evidence from this study to suggest that a complete coating of sediment formed around the small and medium sized microplastics, but that 216 h of abrasion was insufficient to form a complete coating around the large microplastics (figure 7). Although the particle velocities achieved within the chamber were realistic, it is difficult to scale up the results to determine the implications for microplastic exposure in the natural environment. Based on comparison of mineral particle alterations within the abrasion chamber with those observed in the field, estimates suggest that 1−2 h abrasion in the test-tube chamber might represent 1 km of distance travelled in the wind for sub-rounded to rounded quartz [26]. This suggests microplastics would need to be transported 100−200 km in saltation (i.e. within an active particle cloud) to achieve the changes described here.

It was not possible to separate the total number of fragments produced during abrasion into mineral and microplastic components in this study. However, we have added to the body of research exploring the effect of mechanical abrasion on microplastic properties (e.g. [8,67,69]). In particular, the surface physical and chemical properties of the microplastics tested underwent substantial change regardless of which sediments were used as the erodents. These changes can be summarized as an inferred reduction in surface roughness and a reduction in carbon detectable on the microplastic surface. For some combinations of sediment and microplastic (e.g. ROU-Mp_212_) there is a substantial change in the surface chemistry between one period of abrasion and the next (e.g. 72 h and 144 h). For this, and other combinations with large surface chemistry changes it would be valuable to conduct additional experiments to determine whether a distinct change threshold exists.

The effects of environmental exposure on microplastics are expected to vary considerably depending on the nature of the environment (humid or arid) and the effect of physical and chemical weathering [62,70]. The changes to microplastic surfaces described here are unlikely to affect the susceptibility of individual particles to atmospheric transport as the parent plastic particles lie well within the normal size and mass ranges for aeolian particle transport both before and after abrasion. However, the changes to microplastic surface texture described here may have implications for their ability to transport organic pollutants and heavy minerals through the environment [71]. In particular, mechanical abrasion contributes to microplastic ageing which is known to increase the ability of microplastics to adsorb organic contaminants [72]. The subsequent atmospheric transport of microplastics to which contaminants have been adsorbed may facilitate the latter’s distribution to distal locations previously isolated from such substances [73]. Any changes to microplastic surface texture could also affect the properties they contribute to the atmosphere, such as the ability to act as ice-nucleating particles [74] or affect radiation balance [75]. Detailed quantification of microplastic surface roughness could be achieved using techniques such as atomic force microscopy. At a slightly larger scale, given that polyethylene is hydrophobic and quartz sand is hydrophilic, the formation of a quartz crust on the plastic surface may have implications for water adsorption, interparticle bonding and therefore soil crust formation. The concentrations of microplastics on the soil surface required to affect crusting would need to be explored.

## 5. Conclusions

This study demonstrates that microplastics undergo significant surface and chemical transformations during aeolian abrasion, driven by interactions with mineral sediments. Angular erodents, due to their enhanced mechanical properties, generated the highest fragment yields and accelerated changes in microplastic surface chemistry and texture. Larger microplastics amplified abrasion dynamics, influencing particle velocities and fragment production. Surface alterations included roughness reduction, cracking and mineral adhesion, with surface composition analysis highlighting increased oxygen and silicon content on microplastic surfaces. These changes affect microplastic environmental behaviour, including transport, deposition and interactions with ecosystems.

The findings emphasize the need for refined atmospheric models that incorporate microplastic dynamics and erodent interactions. By elucidating the mechanisms of abrasion and their environmental implications, this research provides a foundation for strategies to address microplastic pollution. Future work should explore broader environmental conditions, including varying wind speeds, humidity and mixed particle compositions, to fully capture the complexity of microplastic behaviour in the environment.

## Data Availability

Data provided in a supplementary excel table [76].
